# Supplementary material for: Causal Relationship between Aging and Anorexia Nervosa: A White-Matter-Microstructure-Mediated Mendelian Randomization Analysis
Source: Biomedicines. 2024 Aug 16;12(8):1874. doi: 10.3390/biomedicines12081874 (PMC11351342; doi:10.3390/biomedicines12081874)
Supplement: Supplementary file 1 [file biomedicines-12-01874-s001.zip › Supplementary Figure/Figure S8/Leaveoneout plot of MR analysis for TL to ubm-b-2100.pdf]

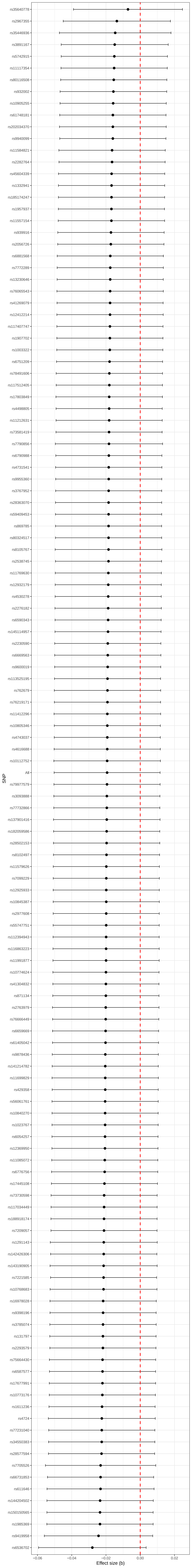
rs35640778
rs2967355
rs35446936
rs3891167
rs5742915
rs11117354
rs80116508
rs932002
rs10905255
rs61748181
rs202034370
rs9940099
rs11584821
rs2282764
rs45604339
rs1332941
rs185174247
rs1957937
rs11557154
rs939916
rs2056726
rs6881568
rs7772289
rs13230646
rs76065543
rs41269079
rs12412214
rs117407747
rs1907702
rs1003322
rs6751209
rs78491606
rs117512405
rs17803849
rs4498805
rs11212631
rs73581419
rs7790856
rs6790988
rs4731541
rs9955360
rs3767952
rs28363070
rs59409453
rs869785
rs80324517
rs8105767
rs2538745
rs11769630
rs12932179
rs4530278
rs2276182
rs6590343
rs145114957
rs2230590
rs6669563
rs9600019
rs113525195
rs762679
rs76219171
rs11412296
rs10805346
rs4743037
rs4616688
rs10112752
A8
rs79977579
rs3093888
rs77732866
rs137901416
rs182059586
rs28502153
rs8102497
rs11579626
rs7099229
rs12925933
rs10845987
rs2977608
rs55747751
rs112394943
rs116863223
rs11991877
rs10774624
rs41304832
rs871134
rs2763979
rs76666449
rs6659669
rs61405042
rs9878436
rs141214782
rs11699829
rs429358
rs56061761
rs10840270
rs1023767
rs6054257
rs12369950
rs11085072
rs6776756
rs17445108
rs73730598
rs117034449
rs188918174
rs7209057
rs1291143
rs142426306
rs143190905
rs7221585
rs10768683
rs16978028
rs9398196
rs3785074
rs131797
rs2293579
rs75664430
rs6587577
rs17677991
rs10773176
rs1611236
rs4724
rs77231040
rs34550383
rs28577594
rs7705526
rs66731853
rs611646
rs144204502
rs150150565
rs1985369
rs9419958
rs6536702
SNP
-0.06
-0.04
-0.02
0.00
0.02
Effect size (b)
